# Pulmonary vein isolation with a pulsed field system leads to lower levels of miR-23a-3p in the left atrial blood compared to a cryoballoon

**DOI:** 10.1038/s41598-025-34187-w

**Published:** 2026-01-11

**Authors:** Andreas Zietzer, Vincent Knappe, Frederick Braun, Philip Düsing, Sophia Grunewald, Ansgar Ackerschott, Laurine Reese, Marko Bulic, Maximilian Funken, Christopher Gestrich, Georg Nickenig, Thomas Beiert

**Affiliations:** Heart Center Bonn, Department of Internal Medicine II, University Hospital Bonn, University of Bonn, Bonn, Germany

**Keywords:** Pulsed field ablation, Cryoballoon ablation, MicroRNA, Atrial fibrillation, Next generation sequencing, Cardiovascular biology, Interventional cardiology

## Abstract

Atrial fibrillation (AF) is the most common arrhythmic disease in humans, with its incidence rising over the past decade. Novel treatment options for AF include pulsed field ablation (PFA) of the pulmonary veins through electroporation. While this technique is thought to be less damaging to surrounding tissue, the acute effects of PFA on the atrium are only incompletely understood. An analysis of circulating microRNAs (miRs) potentially released by local tissue damage due to PFA, in comparison to other standard ablation procedures, has not yet been performed. Small RNA sequencing analysis was performed on left atrial blood samples from four patients after PFA and five patients after cryoballoon ablation (CBA). In this pilot analysis, 154 miRs were stably detected across all samples. The expression of the two miRs with the lowest p-values was validated in 20 PFA cases versus 25 CBA cases via real-time PCR. We found hsa-miR-23a-3p to be significantly less abundant in plasma in the left atrium after PFA compared to CBA. The circulating miR signature in the left atrium is differentially affected by PFA compared to CBA. This finding potentially influences the use of miRs as biomarkers for prediction of outcomes after ablation.

## Introduction

Atrial fibrillation (AF) is the most common arrhythmia in humans. Its prevalence is closely related to age^1^. Recommended therapeutic strategies include rhythm control, aiming to permanently restore sinus rhythm, as opposed to heart rate control, where the rhythm disorder persists^2^. A key element of the rhythm control strategy is the electrical isolation of the pulmonary veins (PVI), which, until recently, has most commonly been achieved through the application of thermal energy in the form of cold (cryoballoon ablation, CBA) or heat through electrical current (radiofrequency ablation)^3^. Within the past years a novel promising ablation technique has been introduced using a pulsed electrical field to electroporate and thereby destroy cardiomyocytes in the left atrium (pulsed field ablation, PFA). PFA has been shown to be comparably effective in isolating pulmonary veins with improved safety regarding two major complications of PVI, namely persisting phrenic nerve injury and atrio-oesophageal fistula^4^. This is thought to be achieved by the higher specificity of this non-thermal energy application to cardiomyocytes^5^.

In recent years, non-coding RNAs have become essential regulators of cellular processes by binding to RNAs, proteins, and DNA. MicroRNAs (miRs), a subset of non-coding RNAs, typically inhibit mRNA translation. Their short length allows miRs to target multiple mRNAs in various tissues, leading to diverse biological effects based on target mRNA expression^6^. In AF, miRs play key roles in pathophysiological processes like atrial fibrosis and electrical remodeling^7^. Circulating miRs have also been suggested as markers for tracking disease progression. Hence, knowledge about the effect of AF treatment on the circulating miR pool is crucial. The effect of PFA on circulating miRs compared to other standard procedures, however, has not yet been investigated.

## Methods

### Patient recruitment, procedures and blood preparation

Patients were recruited at the University Heart Center of Bonn. We included n = 45 consecutive patients undergoing routine first-time pulmonary vein isolation due to symptomatic atrial fibrillation. CBA was performed using the POLARx™ Cryoablation System (Boston Scientific) or the Arctic Front Advance Pro Cryoablation System (Medtronic) following the manufacturer’s instructions as described elsewhere^8^. For PFA, the FARAPULSE—Pulsed Field Ablation System (Boston Scientific) was used according to the manufacturer’s recommendation. During PFA, catheter contact was assessed fluoroscopically by evaluating the back bending of the ablation catheter in basket and flower configuration. For CBA, vein occlusion following contrast injection served as a surrogate for adequate tissue contact. Blood was drawn under sterile conditions immediately at the end of the respective procedure through a steerable sheath in the left atrium. Sodium citrate was used as an anticoagulant, and the blood was processed and stored as previously described to obtain platelet-free plasma^9^. The study complies with the Declaration of Helsinki. All patients gave written and informed consent. The study protocols were approved by the ethics committee of the University Hospital of Bonn (19/425).

### RNA isolation and sequencing

RNA from human plasma was isolated by use of the miRNeasy Serum/Plasma Kit following the manufacturers instructions (Qiagen Cat# 217184). Small RNA sequencing was performed by the NGS-Corefacility of the University Hospital Bonn, for library preparation the NEXTFLEX® Small RNA-Seq Kit v4 was used. Quality control and quantification was performed with the Tapestation 4200 (Agilent). The sequencing was performed in a NovaSeq 6000 and a read length of 1 × 50 bp.

### qPCR analysis

To prepare the cDNA, 10 ng of total RNA was reverse transcribed using the TaqMan microRNA Reverse Transcription kit (Applied Biosystems) following the manufacturer’s instructions. TaqMan microRNA assays from Thermo Fisher Scientific were employed to quantify miRs in a Quant Studio 5 Real-Time PCR instrument (Applied Biosystems). Specifically, hsa-miR-23a-3p (Cat# 4427975, Assay ID 000399), hsa-miR-196b-5p (Cat# 4427975, Assay ID 002366) and U6 snRNA (Cat# 4427975, Assay ID 001937) were analyzed. U6 was used as an internal control and relative miR-levels were calculated as 2^-ΔCT^ and transformed logarithmically as previously reported for plasma miR-levels^10^.

### Statistical analysis

The nf-core/smrnaseq pipeline version 2.2.3 was applied to process the sequence reads and to quantify mature miRs using the human genome GRCh38 and default parameters. The statistical analysis was carried out in the R environment (version 4.2.3). Low abundant miRs with more than 5 counts in less than 3 samples were excluded from the analysis. To identify differentially expressed miRs the DESeq2 package (version 1.38.3) was used to compute the statistical contrast between the two conditions PFA and CBA. For multiple testing correction the Benjamini–Hochberg method was applied and the resulting false discovery rates (FDRs) were reported.

The means of two groups were compared with an unpaired t-test.

## Results

MiR-23a-3p is the most significantly different circulating miR in left atrial blood comparing PFA and CBA.

In our analyses, we included 45 patients, 20 of which were treated with PFA and 25 with CBA. The patients had an average age of 65.64 ± 11.36 years, 18 (40%) of the patients were female. The mean BMI was 27.23 ± 4.59 kg/m^2^ and 19 (42%) of the patients had paroxysmal vs. 26 (58%) persistent AF. Comparison of CVD risk factors revealed no clinically meaningful differences (Table 1). In the PFA group, a history of stroke and peripheral artery disease was more prevalent, leading to higher levels of CHA_2_DS_2_-VASc Score. Creatinine levels as markers of kidney disease were comparable between the two groups. Procedure times were comparable between the two groups, with procedure times for PFA being marginally shorter. The times from the start of the first / last ablation to sampling of the blood in the LA after the procedure were comparable, as well (Table 2).

**Table 1:** Baseline and clinical parameters of the study population.

	Patients (n = 45)	CBA (n = 25)	PFA (n = 20)
Baseline characteristics			
Age [y]	65.64 ± 11.36	64.36 ± 11.64	67.25 ± 11.09
Sex, n (%)			
Female	18 (40)	9 (36)	9 (45)
Male	27 (60)	16 (64)	11 (55)
Body mass index [kg/m^2^]	27.23 ± 4.59	26.93 ± 4.10	27.59 ± 5.23
CHA_2_DS_2_-VASc-Score	2.58 ± 1.96	2.12 ± 1.81	3.15 ± 2.03
LA-Volume [ml]	56.07 ± 26.40	56.74 ± 30.44	56.54 ± 21.02
Laboratory parameters			
Creatinine [mg/dL]	0.97 ± 0.31	0.92 ± 0.16	1.02 ± 0.44
Medical history, n (%)			
Paroxysmal atrial fibrillation	19 (42)	12 (48)	7 (35)
Persistent atrial fibrillation	26 (58)	13 (52)	13 (65)
Smoking	16 (36)	5 (20)	11 (55)
Stroke or TIA	5 (11)	1 (4)	4 (20)
Peripheral artery disease	5 (11)	1 (4)	4 (20)
Coronary artery disease	9 (20)	4 (16)	5 (25)
Arterial hypertension	27 (60)	13 (52)	14 (70)
Diabetes	5 (11)	2 (8)	3 (15)
Hypercholesterolemia	24 (53)	11 (44)	13 (65)

**Table 2:** Periprocedural times.

	Patients (n = 45)	CBA (n = 25)	PFA (n = 20)
Periprocedural times [min]			
Skin to skin	68 ± 13	70 ± 14	66 ± 11
Skin to first ablation	34 ± 9	34 ± 9	35 ± 11
Start first ablation to sampling	30 ± 5	32 ± 3	27 ± 6
Start last ablation to sampling	14 ± 5	12 ± 4	16 ± 5

**Fig. 1:**
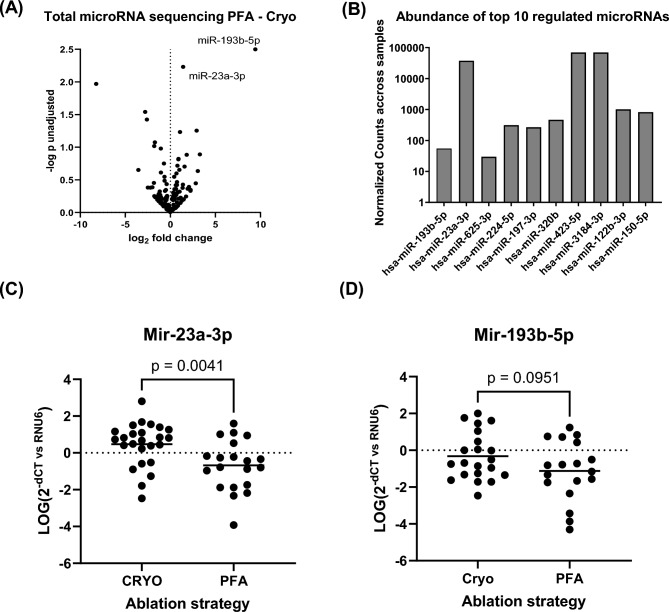
(**A**) Small RNA sequencing analysis of left atrial plasma from patients directly after PFA vs CBA n = 4/5. (**B**) Abundance of detected miRs in all 9 samples. (**C**, **D**) Single rt qPCR analysis of miR-23a-3p and miR-193b-5p expressed as relative levels normalized to U6 n = 25 / 20. All data are presented as individual experiments with the mean; unpaired t-Test.

Initially, we performed an unbiased approach utilizing plasma from four patients in the PFA group compared to five patients in the CBA group for small RNA sequencing. We identified 154 miRs, that were consistently detectable across all samples. While there were numerical variances in the expression levels of certain miRs, no significant differences were detected after adjusting for multiple comparisons in our pilot cohort (Fig. 1A,B). However, the two miRs with the lowest p-values were used for further analysis in a larger cohort of patients. We observed significantly lower levels of miR-23a-3p and in the left atrial blood immediately following PFA compared to CBA (Fig. 1C). This finding aligns with the trend observed in the initial small RNA sequencing analysis. MiR-196b-5p showed a similar trend, but circulating miR-196b-5p was far less abundant and not detectable in 6 CBA and 2 PFA patients (Fig. 1D). This may explain the lack in statistical difference.

## Discussion

In this manuscript, we present a pioneering investigation into the differential expression of miRs in the left atrial blood of patients undergoing PVI using PFA versus CBA. To our knowledge, this is the first comprehensive analysis that compares miR expression patterns in these two ablation modalities. Our results reveal that PFA is associated with significantly lower levels of miR-23a-3p in the left atrial blood immediately after the procedure when compared to CBA.

CBA has been extensively adopted worldwide and is recognized as a standard and effective first-line treatment for atrial fibrillation (AF) ablation, as evidenced in clinical guidelines and its widespread use in clinical practice^3,11^. In the ADVENT trial, CBA (together with RF ablation) served as a comparator arm to PFA; however, direct comparisons between PFA and CBA, particularly those evaluating specific clinical endpoints such as procedural efficacy and tissue injury, are still limited and ongoing^4,11^. This study addresses a knowledge gap by focusing on circulating miR patterns as potential biomarkers of tissue response to these ablation modalities.

MiR-23a-3p is a relevant mediator of cardiac tissue remodeling and atrial fibrillation. It has been shown to promote cardiac hypertrophy by suppressing the transcription factor Foxo3a, forming a regulatory axis that modulates oxidative stress and hypertrophic signaling^12^. Furthermore, miR-23a-3p exacerbates ferroptosis by targeting SLC7A11 and thereby influences the progression of atrial fibrillation^13^.

Our analysis identifies significantly lower plasma levels of miR-23a-3p following PFA compared to CBA. According to the microRNA TissueAtlas 2025 by Rishik et al., miR-23a-3p is predominantly expressed in connective tissue cells. This observation suggests that the higher levels of miR-23a-3p observed following CBA may reflect a greater degree of collateral impact on non-cardiomyocyte cell types, such as fibroblasts, compared to PFA. This would align with previous observations of higher specificity of PFA to cardiomyocytes^14^. Additionally, as with other forms of energy, lesion size in PFA also depends critically on tissue contact and the repetition of applications^15^. Although direct histological assessment of atrial tissue to verify this hypothesis was not performed in this study, higher levels of miR-23a-3p after CBA may have implications for procedural safety and could potentially influence long-term clinical outcomes^16^. Previous studies have predominantly investigated the effect of radiofrequency ablation (RFA) on blood levels of miRs. One study found that RFA increases plasma levels of miR-26 and reduces the expression of P-selectin, suggesting that miR-26a/b may influence AF progression and treatment response^17^. Furthermore, blood levels of miR-21 were shown to correlate with left atrial scarring on an electroanatomical map and predicted worse outcomes after RFA in patients with persistent AF^18^. A cross-comparison of different energy forms and ablation platforms is therefore of high interest to understand how these factors influence circulating miR levels after pulmonary vein isolation, and how this translates into outcomes such as AF recurrence. These questions, however, lie beyond the scope of this pilot investigation and will be addressed in upcoming trials. In those studies, it would also be of interest to evaluate how miR profiles change during the procedure.

### Limitations

This study has several limitations that should be acknowledged. At baseline we observed differences in patient characteristics, such as the prevalence of stroke and peripheral artery disease resulting in a slightly higher CHA_2_DS_2_-VASc-Score. These differences could influence the observed miR patterns and complicate direct comparisons between the PFA and CBA groups. This is particularly the case, because miR-23a blood levels have been shown to be influenced by vascular diseases^19^. Here, randomization of the procedures could be an important approach for upcoming studies. Furthermore, the small sample size may limit the generalizability of our findings, and the cross-sectional nature of the analysis introduces potential biases, including incomplete control over confounding variables. The evaluation of miR blood levels was performed at a single time point, which does not allow for assessment of their kinetics. Despite these limitations, the study’s strength lies in its novel approach—being the first to perform a comprehensive analysis of circulating miRs in the left atrial blood directly following PFA and CBA.

## Conclusion

The circulating miR profile in the left atrium is distinctly altered by PFA compared to CBA. This discovery may have important implications for understanding atrial tissue biology and for utilizing miRs as biomarkers to predict AF recurrence following ablation. The study provides valuable insights into the biological impact of these ablation techniques and underscores the potential of miR profiling as a tool for advancing our understanding of procedural outcomes.

## Data Availability

The datasets generated and analysed during the current study are available in the EMBL platform ArrayExpress under the accession number E-MTAB-14837: https://www.ebi.ac.uk/biostudies/arrayexpress/studies/E-MTAB-14837?key=52e965d2-f5c2-4124-9249-178668e4567e
